# Irreducible Metacarpal Neck Fracture due to Interposition of the Extensor Tendons

**DOI:** 10.1055/s-0041-1731656

**Published:** 2021-10-01

**Authors:** Sathya Vamsi Krishna, Rajendra Kumbar, Sagar Vishwanath Kura, Soumen Das De

**Affiliations:** 1 Departamento de Cirurgia da Mão, Instituto de Ortopedia e Trauma, Sanjay Gandhi, Byrasandra, Jayanagar, Bangalore, India; 2 Departamento de Microcirurgia Reconstrutiva e Cirurgia da Mão, National University Health System, Singapore

**Keywords:** fractures, bone, fracture fixation, internal, metacarpal bones, tendons

## Abstract

A 15-year-old male presented with multiple right-hand fractures, including a severely angulated small finger metacarpal neck fracture. Multiple closed attempts failed, and open reduction was performed. At surgery, the extensor tendon was found to be interposed within the fracture, thereby preventing closed reduction. The tendon was extricated from the fracture site, adequate reduction was obtained, and the fracture was stabilized using K-wires. The fracture united well, with good return of motion and strength. Extensor tendon interposition is a rare scenario associated with metacarpal neck fractures and should be suspected when there is complete loss of contact between the fracture ends and multiple attempts at closed reduction have failed.

## Introduction

Metacarpal fractures comprise 30% of all hand fractures, and the small and ring metacarpals are the most commonly affected. 1 Fractures of the small finger metacarpal neck – also known as “boxer's fracture”– are frequently caused by a direct impact on the fully flexed metacarpophalangeal joint (MPJ). 2 Although boxer's fractures have good healing potential, the relative indications for surgery include rotational deformity, angulation > 60°, and pseudoclawing of the digit. Although it is unusual, the inability to obtain closed reduction or the occurrence of nonunion in these fractures should always raise suspicion of interposition of surrounding structures within the fracture site, mandating intervention. In the present report, we present a rare case of irreducible 5 ^th^ metacarpal neck fracture due to an interposed extensor tendon that required open reduction.

## Case Report

**Fig. 1:**
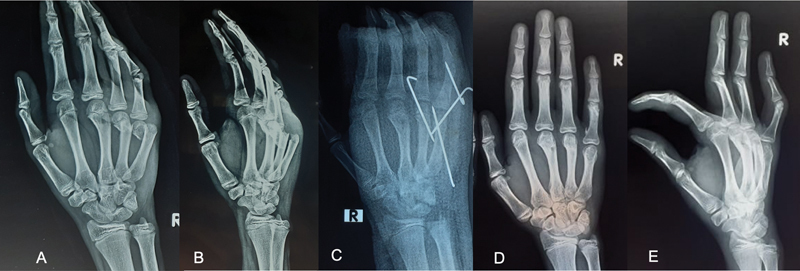
( **A,B** ) Anteroposterior and oblique radiographs of the affected hand showing juxtaphyseal fracture on the neck of the fourth and fifth metacarpals; ( **C** ) Postoperative radiographs of the affected hand following fracture fixations with multiple Kirschner wires; ( **D/E** ) Follow-up radiographs of the hand at 5 months revealing a well-united fracture of the 5 ^th^ metacarpal neck.

**Fig. 2:**
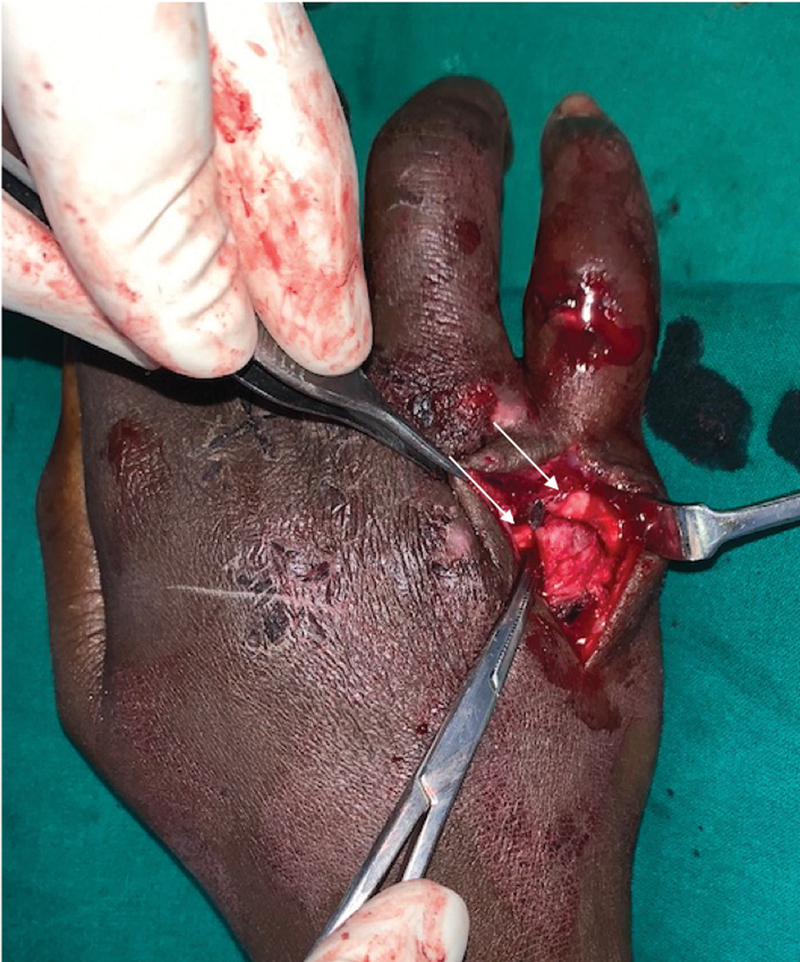
Intraoperative picture showing fracture of the 5 ^th^ metacarpal with the interposed extensor tendons (marked by white arrow marks).

A 15-year-old male presented with multiple hand fractures after a motor vehicle accident. On examination, there was loss of the metacarpophalangeal joint (MPJ) (“knuckle”) prominences of the small and ring fingers with swelling and tenderness over these areas. There was a puncture wound over the dorsum of the small finger proximal interphalangeal joint (PIPJ). Radiographs showed fractures of the ring and small finger metacarpal necks and an intra-articular fracture of the head of the proximal phalanx of the small finger ( Fig. 1 ). The small finger metacarpal was angulated > 60°, with minimal contact between the bone ends. Surgical treatment of the injuries was performed. First, the small finger wound was debrided, and the head of proximal phalanx was fixed with a single 0.045-inch Kirschner wire (K-wire). The small finger metacarpal neck fracture was manipulated multiple times using the Jahss maneuver, but closed reduction was not possible, and the fracture site was exposed via a dorsal midline incision. We found that the proximal fragment was displaced dorsally and that the extensor digiti quinti minimi (EDQM) and the extensor digitorum communis of the small finger (EDC-IV) were interposed between the fracture fragment, concealing the distal fragment ( Fig. 2 ). The juncturae tendinum was divided on the radial side, and the ulnar sagittal band was incised so that the tendons could be extricated. The head of the metacarpal was reduced and fixed with 2 crossed 0.045-inch K-wires ( Fig. 1 ). The ring finger metacarpal neck fracture was reduced using a closed technique and was stabilized using percutaneous K-wires. The hand was immobilized in a thermoplastic splint for 4 weeks in an intrinsic-plus position. After this period, the K-wires were removed and intermittent active motion was initiated. The fractures united in a timely manner and the patient returned to all pre-injury activities at 3 months ( Fig. 1 ).

## Discussion

In children, metacarpal neck fractures of the small and ring fingers are common due to the metacarpal geometry. The metacarpal angulates distally toward the MCPJ, and the cortical bone is known to be thin within the subcondylar fossa, making it susceptible to injury. These juxtaphyseal fractures are analogous to adult boxer's fracture and have good healing potential. 3 Most 5 ^th^ metacarpal neck fractures with angulation < 40° can be managed conservatively with ulnar gutter splint by immobilizing the wrist, the MCPJ, and the PIPJ in functional position for 3 weeks followed by gentle range of motion. However, fractures with increased angulation, pseudoclawing and rotational deformities are reduced by the Jahss maneuver, and the fractures can be stabilized by various techniques. Malunion is the most commonly known complication leading to loss of knuckle prominence, and nonunion is rare. Requirement of open reduction is exceptional for these fractures unless there is a difficulty in reduction or trouble in correcting the angulation. In our case, we had to perform open reduction since closed maneuver failed to achieve reduction due to interposition of the EDC-IV and the EDQM between the fracture. The probable mechanism could be due to a circumferential injury of the periosteum at the fracture site and buttonholing of the proximal fragment between the tendons and the sagittal band. If this were neglected, there could have been a consequence of definitive fracture nonunion. Tendon interposition is a rare presentation with very few case reports. Blohm et al 4 described a similar presentation with tendon and juncturae tendinum interposition, and Joshy et al. 5 reported a case of boxer's fracture nonunion due to extensor digitorum tendon hindrance.

Although it is rare, the interposition should be anticipated in a Boxer's fracture whenever there is an obvious gap on a radiograph with complete loss of contact at the fracture ends and difficulty in getting adequate closed reduction. Surgeons should be prepared for a subsequent open reduction. With these measures, nonunion can be avoided, and early functions of hand can be restored.

Statement of Informed Consent: The parents of the patient were informed that data concerning the case would be submitted for publication, with which they completely agreed and consented. The present study is exempted from Ethical Committee approval.
