# Supplementary material for: Irreducible Metacarpal Neck Fracture due to Interposition of the Extensor Tendons
Source: Rev Bras Ortop (Sao Paulo). 2021 Oct 1;59(Suppl 1):e9–e11. doi: 10.1055/s-0041-1731656 (PMC11254442; doi:10.1055/s-0041-1731656)
Supplement: Related texts

# Research article: Fratura irredutível do colo do metacarpo devido a interposição dos tendões extensores

**Keywords:** fixação interna de fraturas, fraturas ósseas, ossos metacárpicos, tendões

## Abstract

Um jovem do sexo masculino com 15 anos de idade apresentou múltiplas fraturas da mão direita, incluindo fratura do colo do quinto metacarpo com grande angulação. As múltiplas tentativas de redução fechada falharam, sendo então realizada a redução aberta. Foi encontrado durante a cirurgia o tendão extensor interposto dentro da fratura, impedindo desta forma a redução fechada. O tendão foi liberado do sítio da fratura, de modo que foi obtida a redução adequada e a fratura foi estabilizada com fios de Kirschner. A fratura foi bem consolidada, com um bom retorno do movimento e da força. A interposição do tendão extensor é um cenário raro associado a fraturas do colo metacárpico, devendo se suspeitar dela quando houver perda completa de contato entre as extremidades da fratura e as várias tentativas de redução fechada falharem.

## Introdução

As fraturas metacárpicas são responsáveis por 30% de todas as fraturas da mão, sendo os metacarpos mínimos e anelares os mais comumente acometidos. 1 A fratura do colo do quinto metacarpo – também conhecida como a “fratura do pugilista” – é frequentemente causada por um impacto direto na articulação metacarpofalângea (MPJ) totalmente flexionada. 2 Embora as fraturas do pugilista tenham bom potencial de consolidação, as indicações relativas para a cirurgia incluem deformidade rotacional, angulação > 60° e a formação de dedo em garra. Ainda assim, a incapacidade de obter uma redução fechada ou a ocorrência de pseudoartrose nessas fraturas devem sempre levantar suspeitas sobre a interposição das estruturas circundantes dentro do sítio da fratura, consequentemente exigindo uma intervenção. No presente relato, apresentamos um caso raro de fratura irredutível do colo do quinto metacarpo, devido a um tendão extensor interposto que necessitou de redução aberta.

## Relato de Caso

Um jovem de 15 anos apresentou múltiplas fraturas na mão após um acidente com veículo motorizado. No exame físico, havia perda das proeminências da articulação MPJ (“nódulos”) dos dedos mínimos e anelares, apresentando edema e sensibilidade nessas áreas. Havia um ferimento por punção no dorso da articulação interfalângica proximal do dedo mínimo (PIPJ). As radiografias mostraram fratura do colo metacárpico do dedo mínimo e anelar, além de uma fratura intra-articular da cabeça da falange proximal do dedo mínimo ( Fig. 1 ). O metacarpo do dedo mínimo foi angulado além de 60° com contato mínimo entre as extremidades ósseas. Foi realizado tratamento cirúrgico das lesões. Primeiramente, a ferida do dedo mínimo foi desbridada e a cabeça da falange proximal foi fixada com um único fio de Kirschner de 0,045 polegadas (fio K). A fratura do colo metacárpico do dedo mínimo foi manipulada várias vezes, por meio da manobra de Jahss, porém a redução fechada não foi possível e o sítio da fratura foi exposto, mediante uma incisão na linha média dorsal. Verificamos que o fragmento proximal foi deslocado dorsalmente e o extensor digiti quinti minimi (EDQM) e o extensor digitorum communis (EDC-IV) estavam interpostos ao dedo mínimo entre o fragmento da fratura, ocultando o fragmento distal ( Fig. 2 ). A junção tendínea foi seccionada no lado radial e a banda sagital ulnar foi incisada, a fim de que os tendões pudessem ser liberados. A cabeça do metacarpo foi reduzida e fixada com 2 fios K cruzados de 0,045 polegadas ( Fig. 1 ). A fratura do colo metacárpico do dedo anelar foi reduzida pela técnica fechada, sendo estabilizada com fios K percutâneos. A mão foi imobilizada com uma tala termoplástica durante 4 semanas, em posição intrínseco plus. Depois deste período, os fios de K foram removidos, sendo iniciado o movimento ativo intermitente. As fraturas se consolidaram em um curto período, e o paciente retornou a todas as atividades pré-lesão em 3 meses ( Fig. 1 ).

**Fig. 1:** ( **A, B** ) Radiografias anteroposterior e oblíqua da mão lesionada, mostrando fratura justafisária do colo do quarto e quinto metacarpos; ((C)) radiografias pós-operatórias da mão lesionada, após fixação da fratura com diversos fios de Kirschner; ( **D/E** ) Radiografias de acompanhamento da mão aos 5 meses, revelando uma fratura bem consolidada do colo do quinto metacarpo.

**Fig. 2:** Imagem intraoperatória mostrando fratura do quinto metacarpo com os tendões extensores interpostos (marcados por setas brancas).

## Discussão

Nas crianças, as fraturas do colo metacárpico do dedo mínimo e anelar são comuns, devido à geometria do metacarpo. O metacarpo angula distalmente em direção à articulação MF e o osso cortical, que é conhecido por ser fino dentro da fossa subcondilar, tornando-o suscetível a lesões. Essas fraturas justafisárias são análogas às fraturas do pugilista adulto, apresentando um bom potencial de consolidação. 3 A maioria das fraturas do quinto colo metacárpico com angulação < 40° pode ser tratada de forma conservadora, com tala de calha ulnar, imobilização do punho e das articulações MF e IFP em posição funcional durante 3 semanas, seguidas de amplitude de movimento suave. No entanto, as fraturas com angulação aumentada, as pseudogarras e as deformidades rotacionais são reduzidas pela manobra descrita por Jahss, podendo ser estabilizadas por várias técnicas. As fraturas viciosamente consolidadas são a complicação mais comumente conhecida, levando à perda da proeminência dos nódulos. A pseudoartrose é rara. A necessidade de redução aberta é excepcional para essas fraturas, a menos que exista dificuldade na redução ou na correção da angulação. Tivemos que realizar a redução aberta no nosso caso, já que a manobra fechada não conseguiu reduzir, devido à interposição dos tendões EDC-IV e EDQM entre a fratura. O provável mecanismo pode ser decorrente da lesão circunferencial do periósteo no sítio da fratura e o dedo em botoeira do fragmento proximal entre os tendões e a banda sagital. Caso seja negligenciada, pode ter uma consequência definitiva de pseudoartrose da fratura. A interposição do tendão é uma manifestação rara, com poucos relatos de caso. Blohm et al. 4 descreveram uma apresentação semelhante com a interposição do tendão e da juncturae tendinum. Joshy et al. 5 relataram um caso de não consolidação da fratura do pugilista devido à obstrução do tendão extensor digitorum.

Embora rara, a interposição deve ser antecipada em uma fratura de pugilista, sempre que houver um espaço óbvio em uma radiografia, com perda completa de contato nas extremidades da fratura e dificuldade em obter uma redução fechada adequada. Os cirurgiões devem estar preparados para uma redução aberta subsequente. Com essas medidas, pode ser evitada a falha na consolidação e as funções anteriores da mão podem ser restauradas.

Termo de Consentimento Livre e Esclarecido: Os pais do paciente foram informados de que os dados relativos ao caso seriam submetidos para publicação, com a qual concordaram e consentiram totalmente. O presente estudo está isento de aprovação pelo Comitê de Ética.
